# Consideration of possible effects of vitamin D on established cancer, with reference to malignant melanoma

**DOI:** 10.1111/pcmr.13040

**Published:** 2022-05-11

**Authors:** Peter E. Hutchinson, James H. Pringle

**Affiliations:** ^1^ Department of Dermatology Leicester Royal Infirmary Leicester UK; ^2^ Leicester Cancer Research Centre University of Leicester Leicester UK

**Keywords:** anti‐tumour immunity, melanoma progression, vitamin D, vitamin D receptor, vitamin D signalling

## Abstract

Epidemiological studies indicate that Vitamin D has a beneficial, inhibitory effect on cancer development and subsequent progression, including melanoma (MM), and favourable MM outcome has been reported as directly related to vitamin D_3_ status, assessed by serum 25‐hydroxyvitamin D_3_ (25[OH]D_3_ ) levels taken at diagnosis. It has been recommended that MM patients with deficient levels of 25(OH)D_3_ be given vitamin D_3_ . We examine possible beneficial or detrimental effects of treating established cancer with vitamin D_3_. We consider the likely biological determinants of cancer outcome, the reported effects of vitamin D_3_ on these in both cancerous and non‐cancerous settings, and how the effect of vitamin D_3_ might change depending on the integrity of tumour vitamin D receptor (VDR) signalling. We would argue that the effect of defective tumour VDR signalling could result in loss of suppression of growth, reduction of anti‐tumour immunity, with potential antagonism of the elimination phase and enhancement of the escape phase of tumour immunoediting, possibly increased angiogenesis but continued suppression of inflammation. In animal models, having defective VDR signalling, vitamin D_3_ administration decreased survival and increased metastases. Comparable studies in man are lacking but in advanced disease, a likely marker of defective VDR signalling, studies have shown modest or no improvement in outcome with some evidence of worsening. Work is needed in assessing the integrity of tumour VDR signalling and the safety of vitamin D_3_ supplementation when defective.

## 1 INTRODUCTION

**FIGURE 1:**
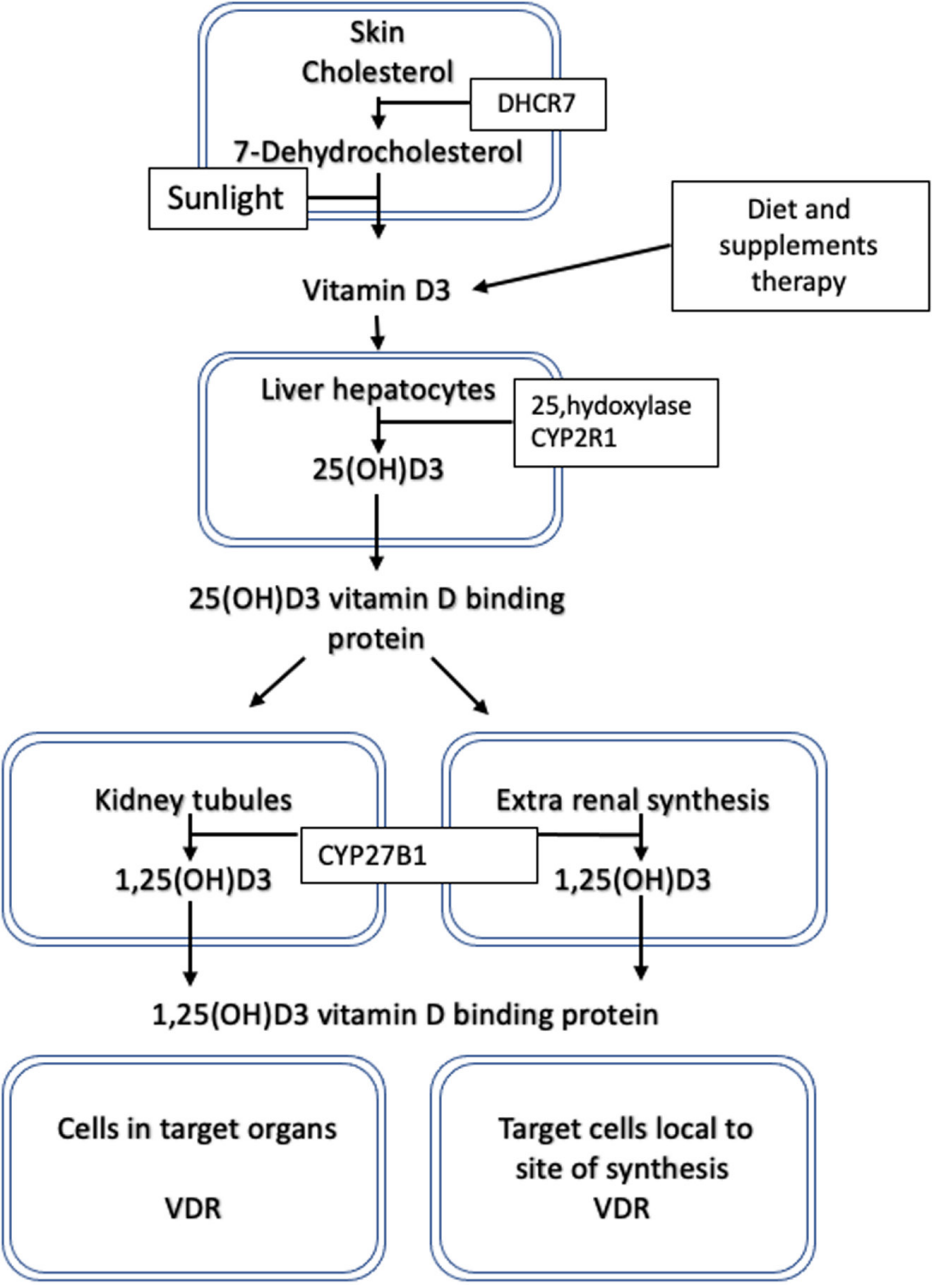
Vitamin D metabolism pathway. In the skin, 7‐dehydrocholesterol is converted into pre‐vitamin D_3_ by UV light and then modified into vitamin D_3_. The dietary or therapeutic sources of vitamin D are transported in the blood by means of vitamin D binding proteins and are hydroxylated in the liver into 25‐hydroxyvitamin D_3_. 25(OH)D_3_ is further hydroxylated in the renal tubules into 1,25 dihydroxyvitamin D_3_, the active form of the hormone. 1,25(OH)_2_D_3_ can also be synthesised in extra renal tissues and cells where it usually acts on local cells as a paracrine or intracrine factor. The amount of 1,25(OH)_2_D_3_ produced in the kidney is tightly regulated by serum calcium, parathyroid hormone and 25(OH)D_3_ levels which control the homeostasis of extracellular fluid (ECF) levels of calcium and phosphate

Vitamin D_3_ status in the body is dependent on the amount of vitamin D_3_ consumed in the diet or synthesised in the skin following sun exposure. Vitamin D_3_ requires activation and is hydroxylated twice, classically, first in the liver to produce 25(OH)D_3_ by 25 hydroxylation and then primarily in the kidney or in immune cells such as macrophages and dendritic cells where the enzyme 25‐hydroxyvitamin D‐1α‐hydroxylase (CYP27B1) converts 25(OH)D_3_ to the active form 1α,25‐dihydroxyvitamin D_3_ (1,25(OH)_2_D_3_). The amount of 1,25(OH)_2_D_3_ produced in the kidney is tightly regulated by serum calcium, parathyroid hormone and 25(OH)D_3_ levels and controls the homeostasis of extracellular fluid (ECF) levels of calcium and phosphate (Morris & Anderson, 2010). The pathway controlling the activation of vitamin D is shown in Figure 1.

An alternative pathway for producing biologically active D_3_‐hydroxyderivatives is via CYP11A1 which hydroxylates the side chain of vitamin D_3_ at carbons 17, 20, 22 and 23 to produce at least 10 other metabolites, with 20(OH)D_3_, 20,23(OH)_2_D_3_, 20,22(OH)_2_D_3_, 17,20(OH)_2_D_3_ and 17,20,23(OH)_3_D_3_ being the main products (Slominski, Kim, et al., 2012; Slominski, Kim, et al., 2015; Slominski, Kim, Li, et al., 2014; Slominski, Kim, Shehabi, et al., 2014; Slominski, Li, et al., 2015). Intermediates are detectable in serum. (Jenkinson et al., 2021; Slominski, Kim, et al., 2015) CYP11A1 is also expressed in the immune system and skin (Slominski, Kim, Shehabi, et al., 2014; Slominski, Tuckey, et al., 2020) and its metabolites have anti‐melanoma activities (Slominski, Brożyna, et al., 2018; Slominski, Janjetovic, et al., 2012). However, CYP11A1 does not act on 25(OH)D_3_ (Slominski, Kim, Li, et al., 2014). Therefore, it is unlikely that these biologically active D3‐hydroxyderivatives are important when considering administration of oral vitamin D_3_ which is rapidly metabolised to 25(OH)D_3_ in the liver.

1,25(OH)_2_D_3_ is a ligand for the vitamin D receptor (VDR) which acts in combination with the retinoid X receptor (RXRA) to regulate transcription of many genes by binding to vitamin D receptor response elements, (VDREs) in the gene. There are also alternative nuclear receptors for vitamin D hydroxyderivatives with their own response elements (Slominski, Chaiprasongsuk, et al., 2020) including retinoic acid receptor‐related orphan receptors (RORα (NR1F1) and RORγ (NR1F3)) (Slominski, Kim, Takeda, et al., 2014), the aryl hydrocarbon receptor (AhR) (Slominski, Kim, et al., 2018) and the liver X receptor beta (LXRβ (NR1H2) (Slominski et al., 2021). There are reports of these receptors suppressing tumour progression, e.g in MM LXRβ (Pencheva et al., 2014; Zhang, Jiang, Zhang, et al., 2014), AhR (Contador‐Troca et al., 2015) and RORα and RORγ (Brozyna et al., 2016) (note vitamin D_3_ hydroxy products are reverse agonists of RORα and RORγ; Slominski et al., 2017; Slominski, Kim, Takeda, et al., 2014) but they can also have a tumour promoting effect e.g. LXRβ (Nelson et al., 2013), AhR (Su et al., 2013). As mentioned above the relevant hydroxy product here is 1,25(OH)_2_D_3_ which is a ligand of these alternative receptors, but we were unable to find evidence of an effect on tumour growth or anti‐tumour immunity of these receptors with 1,25(OH)_2_D_3_ as ligand. A further point of uncertainty is whether these receptors persist after the VDR in advanced cancer, loss of signalling being central to our argument about a possible deleterious effect of vitamin D_3_ supplements in advanced cancer. We will therefore concentrate on VDR signalling.

The classic roles of vitamin D_3_ are the regulation of calcium uptake, calcium homeostasis, bone metabolism, cell growth, division and differentiation. The last two are potentially beneficial in controlling tumour cell growth. However, the expression of VDR has been identified in many tissues in different cell types and the action of 1,25(OH)_2_D_3_ has important implications for regulating the immune system, where most cells express VDR, potentially influencing tumour immune surveillance.

Prediagnostic vitamin D_3_ status has a well‐documented protective effect on the development and subsequent progression of cancer, reviewed by Grant (2018). Post‐diagnosis serum 25(OH)D_3_ levels have shown an inverse relation with progression in a number of cancers (Vaughan‐Shaw et al., 2017). An interpretation of this is that vitamin D_3_ has a beneficial effect on established cancer (Newton‐Bishop et al., 2009; Nurnberg et al., 2009). The National Institute for Health and Care Excellence (NICE) recommendations on vitamin D_3_ and MM are to measure 25(OH)D_3_ levels at diagnosis in secondary care in all patients with MM and to give those, whose levels are thought to be suboptimal, advice on vitamin D_3_ supplementation and monitoring in line with local policies and NICE guidelines on vitamin D_3_ (The National Institute for Health and Care Excellence, 2015; Nice Guideline NG14 July 2015 Melanoma: Assessment and Management).

We consider possible beneficial or deleterious effects of vitamin D_3_ administration in established cancer and the possible circumstances dictating a positive or negative effect on outcome. First, we discuss basic determinants of cancer outcome that is, intrinsic tumour aggressiveness, in terms of cancer cell growth, differentiation and migration; associated inflammation; anti‐tumour immune response and angiogenesis, and the likely impact of vitamin D_3_ status and the integrity of VDR signalling in the tumour. We then consider the experimental in vivo, epidemiological and clinical evidence of the effect of vitamin D_3_ in cancer.

## 2 POSSIBLE MECHANISMS OF AN EFFECT OF VITAMIN D3 ON CANCER

### 2.1 Inhibition of tumour cell growth

Vitamin D_3_ has a well‐known inhibitory effect on cell growth, through anti‐proliferative, pro‐apoptotic and anti‐cell migratory activity as reviewed by Fleet et al. ( 2012), Samuel & Sitrin (2008). The effects of vitamin D_3_ on growth are mediated by the action of 1,25(OH)_2_D_3_ on the intracellular VDR, which is a transcription factor. In vitro studies show that vitamin D_3_ inhibits growth in some malignant cell lines (Fleet et al., 2012), including MM (Colston et al., 1981) and promotes differentiation (Samuel & Sitrin, 2008). Moreover, inhibition of experimental carcinogenesis by dietary vitamin D_3_ supplementation and 1,25(OH)_2_D_3_ administration has been demonstrated in vivo in animal models (Beaty et al., 1993; Wood et al., 1983).

These beneficial effects are largely the result of nuclear VDR signalling (Carlberg & Campbell, 2013). Using low nuclear VDR concentration as a marker of defective VDR signalling, 1,25(OH)_2_D_3_ fails to disrupt growth and produce cell death in culture (Hutchinson et al., 2018). Moreover, in tumours with known outcome, histological evidence of low nuclear VDR is associated with progression and metastasis (Brozyna et al., 2011, 2014; Hutchinson et al., 2018).

### 2.2 Suppression of inflammation

Inflammation has been long recognized as oncogenic but, more importantly here, a promotor of tumour progression (Mantovani et al., 2008), including metastasis (Mantovani, 2009). There is evidence, experimental and observational, that vitamin D_3_ suppresses inflammation. Vitamin D_3_ downregulates macrophages in terms of recruitment (Riek et al., 2014) and inflammatory cytokine production (Guillot et al., 2010) such as C‐reactive protein (CRP), interleukin (IL) IL1A, IL1B, IL6, IL8, tumour necrosis factor (TNF), while upregulating anti‐inflammatory cytokines such as IL10 (Guillot et al., 2010). The growth hormone midkine (MDK) is involved in leukocyte recruitment to the sites of inflammation and expression of proinflammatory cytokines and the expansion of regulatory T‐cells as reviewed by Weckbach et al. (2011). A suggested proinflammatory mechanism is the known upregulation of nuclear factor kappa B kinase (NF‐ΚB) (Cerezo‐Wallis et al., 2020). Other relevant effects of MDK in cancer are promotion of angiogenesis (Muramaki et al., 2003), upregulation of integrin mediated cell migration (osteoblast‐like cells) and, through Notch2 binding, induction of epithelial mesenchymal transition (EMT) (immortalized HaCaT keratinocytes). There are no reports of an effect of vitamin D_3_ on MDK in cancer, but this seems feasible as higher levels of MDK are reported in vitamin D deficiency (Serinkan Cinemre et al., 2016). NF‐ΚB is a key transcription factor involved in inflammatory cell differentiation and inflammatory cytokine expression (Liu et al., 2017). The VDR physically interacts with Inhibitor of NF‐ΚB subunit Beta (IKBKB) to block NF‐ΚB activation (Chen et al., 2013). In addition, observational studies in healthy individuals have shown an inverse relation between serum 25(OH)D_3_ and inflammatory markers (Liefaard et al., 2015). Thus, there is good evidence that vitamin D_3_ is anti‐inflammatory which would be expected to be beneficial in all stages of cancer and irrespective of tumour VDR signalling.

### 2.3 Suppression of anti‐tumour immunity

**FIGURE 2:**
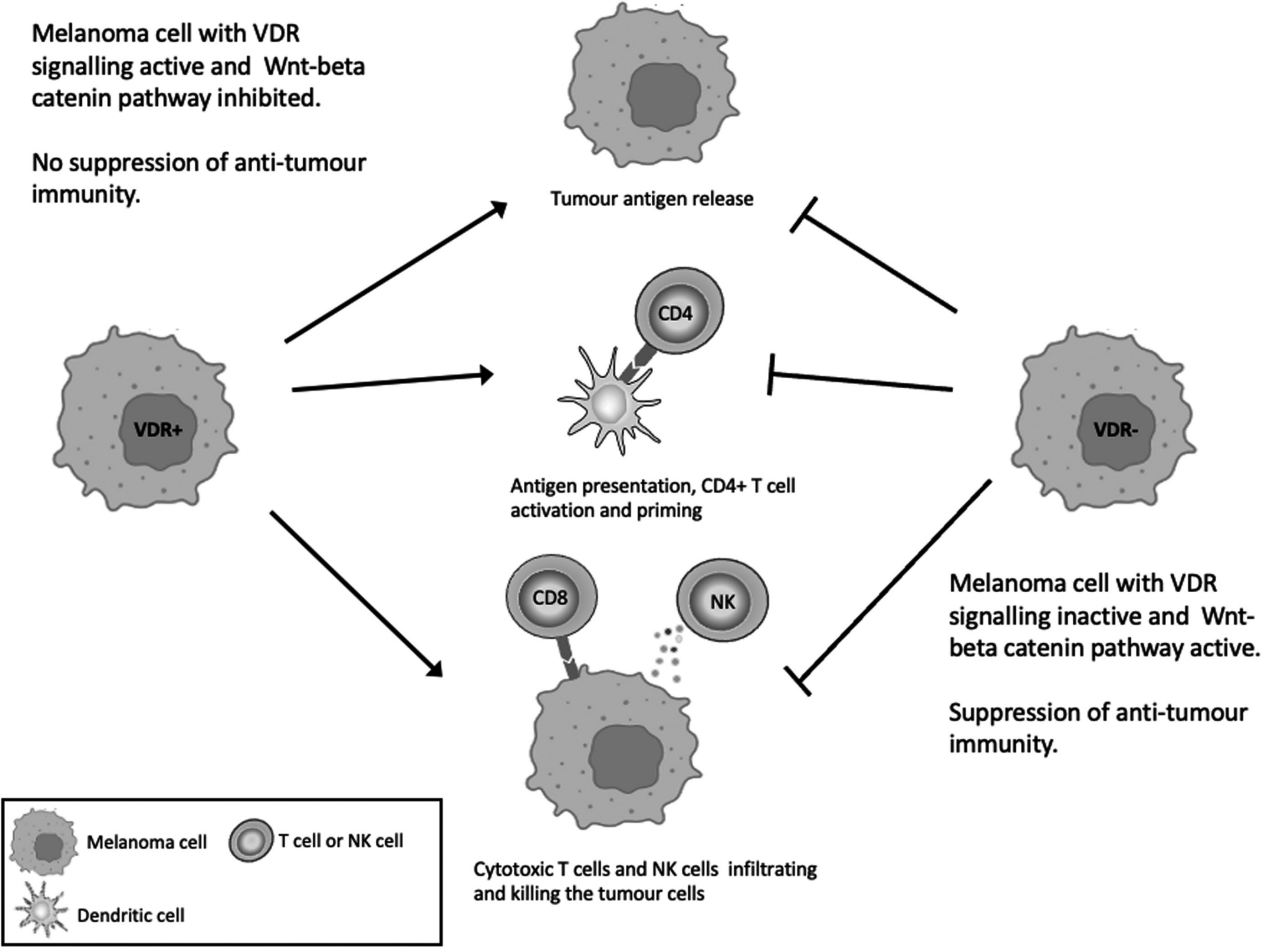
Indirect actions of vitamin D regulating the immune response to melanoma by inhibiting Wnt‐beta catenin signalling. VDR signalling inhibits Wnt‐beta catenin signalling which regulates the tumour‐immune response. There is significant evidence showing that in melanoma Wnt‐beta catenin signalling blocks immune recognition of the tumour at all stages, including tumour antigen release, antigen presentation, T‐cell priming, activation and infiltration as well as tumour cell elimination

Anti‐tumour immunity is a very important determinant of cancer outcome as evidenced by the success of recent immune‐based therapies (Menon et al., 2016). Vitamin D_3_ has been reported to enhance anti‐tumour immunity by increasing the number of tumour associated immunocytes, via tumour VDR suppression of Wnt‐beta catenin signalling (Muralidhar et al., 2019). There is significant evidence showing that Wnt‐beta catenin signalling blocks immune recognition of the tumour at all stages, including tumour antigen release, antigen presentation, T‐cell priming, activation and infiltration as well as tumour cell elimination (see Figure 2; Luke et al., 2019). However, this is an indirect effect of vitamin D_3_ and would appear dependent on intact intra tumour VDR signalling. Defective VDR signalling would therefore be associated with reduced numbers of immunocytes, which however, unlike the tumour, would retain sensitivity to vitamin D_3_. Considering direct effects of vitamin D_3_ on immunocytes, most immunocytes, including dendritic cells (DCs), CD4+ T cells (T4), CD8+ T cells (T8), γδT cells and macrophages, express the VDR (Baeke et al., 2010; Chen et al., 2005; Hewison et al., 2003; Kreutz et al., 1993; Veldman et al., 2000). Vitamin D_3_ has many direct suppressive effects on immune cells, as evidenced by its protective effect against auto‐immune disease (Goldberg, 1974; Hypponen et al., 2001; Mathieu et al., 1992). When considering the tumour/immunity relationship, the term immunoediting (Dunn et al., 2002) is used. This describes a triphased immunological response to tumours comprising phases of elimination, equilibrium and escape, reviewed by Mittal et al. (2014). In the elimination phase, there is host immunological attack on the tumour, in the equilibrium phase, there is balance between tumour proliferation and immune suppression, while in the escape phase, there is suppression of anti‐tumour immunity allowing the tumour to progress.

#### 2.3.1 Elimination phase

The elimination phase (Mittal et al., 2014) involves innate and adaptive immunity. Critical elements are IFNG secretion and cytolytic capacity of immune cells. An important early source of IFNG is γδT cells (Gao et al., 2003), other sources being natural killer cells (NK) and T cells, antigen‐specific effector T‐helper type 1 (Th‐1), T8 cytotoxic T‐cells (CTLs) and natural killer T cells (NKT) cells. IFNG increases tumour cell immunogenicity, by upregulating components of the major histocompatibility complex (MHC) class I protein and promotes maturation of dendritic cells (DCs), generation of Th1 cells and CTLs and activates cytocidal activity in macrophages. Tumour cells are killed by CTLs, NK, NKT, γδT cells and macrophages, mechanisms including apoptosis inducing molecules ((Fas cell surface death receptor ligand(FASLG), TNF superfamily member 10 (TNFSF10)) and cytolytic molecules (granzyme, reactive oxygen species [ROS]). The immune reaction is triggered by expression of ‘stress’ induced tumour haptens, loss of inhibitory molecules on the tumour and expression of tumour antigens, in context of MHC class I and II molecules (Th‐1 and CTLs respectively) or CD1D (NKT cells). An effect of vitamin D_3_ on IFNG in this situation is not reported but 1,25(OH)_2_ D_3_ is known to inhibit IFNG produced by Vγ9Vδ2 T cells (Chen et al., 2005), differentiating NK cells (Weeres et al., 2014), Th1 cells (Staeva‐Vieira & Freedman, 2002), CTLs (Jeffery et al., 2009) and peripheral blood mononuclear cells (PBMCs; Ragab et al., 2016).

In innate immunity, NK cells are activated by tumour expression of stress‐inducible ligands structurally related to MHC class I, MHC Class I polypeptide‐related sequence (MIC) MICA and MICB (Lopez‐Soto et al., 2015), recognized by NK cell activation receptors such as killer cell lectin‐like receptor K1 (KLRK1). Moreover, killer‐cell immunoglobulin‐like inhibitory receptors respond to MHC class 1 on the tumour cell, the absence of which, through malignant transformation or CTL activity, results in NK cell activation. NK cells lyse tumour cells via granzyme and TNFSF10 and FASLG, secrete cytokines, primarily Th‐1 type cytokines such as IFNG, TNF and granulocyte/ monocyte colony‐stimulating factor (CSF2) which facilitate the activation of T cells and other innate immune mediators (Walzer et al., 2005). The effect of vitamin D_3_ on NK cells in cancer is not reported but 1,25(OH)_2_D_3_ reduced perforin‐mediated cytotoxicity of activated NK cells (from patients with recurrent pregnancy loss), by decreasing activating NK receptors and increasing inhibitory NK cell receptors (Ota et al., 2015). However, vitamin D_3_ increases NK activity in lean mice (Lee et al., 2018).

γδT cells, reviewed by Zhao et al. (2018), are activated by metabolites of the mevalonate pathway (phosphoantigens), accumulated by transformed cells (Gober et al., 2003), and also by stress‐induced tumour haptens. Vγ9Vδ2 T cells are a common form of γδ T cells and have direct cytolytic activity involving perforin/granzyme, TNFSF10 and FASLG and produce IFNG. γδT cells may also have an indirect effect on tumour elimination by activation of Th‐1 lymphocytes, antigen specific T8 cytotoxic cells and T4 cytotoxic cells (Mao et al., 2014). Vitamin D_3_ may have an inhibitory effect as it significantly inhibits, in a dose‐dependent fashion, phospholigand‐induced γδ T cells expansion and IFNG production (Chen et al., 2005).

Natural killer T cells (NKT) (reviewed by Nair & Dhodapkar (2017) have, in general, an αβ T‐cell receptor (TCR) of limited diversity responding to extrinsic and intrinsic lipid antigen presented in relation to CD1D, a non‐polymorphic MHC 1‐like molecule. CD1D can be expressed by antigen presenting cells (APCs) and tumour cells, but not usually solid tumours including MM. Type I NKT (invariate NKT) cells are mainly reported to invoke an anti‐tumour immune response (Nair & Dhodapkar, 2017). Increased frequency of type I NKT cells in blood and in the tumour infiltrate are favorable prognostic indices (Nair & Dhodapkar, 2017). Anti‐tumour type 1 cell activity can involve direct tumour lysis, recruitment and activation of other innate and adaptive immune cells by initiating Th1 cytokine cascade, and regulation of recruited immunosuppressive cells in the tumour microenvironment (TME). In experimental autoimmune encephalomyelitis (EAE), 1,25(OH)_2_D_3_ is protective through an effect on NKT type 1 cells, possibly involving IL4 (Waddell et al., 2015) and this would suggest 1,25(OH)_2_D_3_ induces immunosuppressive activity in these cells (Dankers et al., 2016).

Macrophages polarized to M1 macrophages by inflammatory cytokines, INFG and TNF, secrete inflammatory cytokines, IL6, IL12 and TNF, activating T cells and lyse cancer cells. Macrophages polarized to M2 phenotype have regulatory and wound‐healing properties. Regulatory M2 macrophages have anti‐inflammatory properties and are important in resolving inflammation, producing the immunosuppressive cytokine IL10 while wound‐healing M2 macrophages respond to immune complexes, prostaglandins, apoptotic cells and IL10 to produce to IL4 and arginase activity to stimulate collagen synthesis. 1,25(OH)_2_D_3_ may polarize macrophages to M2 phenotype as described below (Liu et al., 2021).

In acquired anti‐tumour immunity, there is activation of tumour antigen‐specific Th‐1 cells, by tumour antigen presented by either APCs or directly by MHC class II expressing tumour cells. IL12, produced by tumour antigen activated APCs, and IL2 are major drivers of the Th‐1 response, IFNG is a major effector and CTLs and macrophages the effector cells. 1,25(OH)_2_D_3_ is reported to polarize T4 cells away from Th‐1 toward Th‐2 phenotype (Sloka et al., 2011). Moreover, there is evidence 1,25(OH)_2_D_3_ downregulates Th‐1 IFNG production in the presence of IL2 (Staeva‐Vieira & Freedman, 2002). In addition, 1,25(OH)_2_D_3_ may downregulate the Th‐1 response by downregulation of DCs. In vitro, addition of 1,25(OH)_2_D_3_ to DCs caused, through inhibition of NF‐ΚB, inhibition of differentiation and maturation, downregulated expression of MHC‐class II, co‐stimulatory molecules and IL12 (Dong et al., 2005).

CTLs are activated by TCR binding with tumour antigen bound to MHC Class 1 on tumour cells or on professional APCs (cross presentation) (Mittal et al., 2014). Further activation requires co‐stimulatory signals and IL2 induced cell proliferation. CTLs, though expressing VDR, are relatively insensitive to anti‐proliferative responses of VDR than CD4+ cells (Iho et al., 1990). However, vitamin D_3_ inhibits the secretion of IFNG and TNF by the activated CD8+ cells (Lysandropoulos et al., 2011).

Th‐17 cells are reported to have both anti‐tumour and tumour promoting actions (Alizadeh et al., 2013; Yousefi et al., 2015). The mechanisms of anti‐tumour activity include induction of tumour derived cytokines (CXCL9 and 10) which attract Th‐1 cells (Kryczek et al., 2009), and subsequently, CD8+ lymphocytes and NK cells (Asadzadeh et al., 2017). Th‐17 also activates NK cells and macrophages to produce IL12 (Jovanovic et al., 1998). VDR blocks binding of the transcription factor NFAT1 to the promoter of the human IL17 gene leading to a decrease in IL17 production in Th‐17 autoimmunity (Joshi et al., 2011).

Thus, in the absence of tumour VDR signalling, many of the reported immunological effects of vitamin D_3_ might oppose the immunological attack on the tumour in the elimination phase including downregulation of IFNG production and downregulated activity of NK cells, γδT cells, Th‐1 cells, CTLs and Th‐17 cells. It is of note that these are described effects of vitamin D_3_ but not confirmed in cancer.

#### 2.3.2 Equilibrium phase

In this phase, there is a balance between tumour proliferation and apoptosis induced by anti‐tumour immunity. The suppressive action of vitamin D_3_ on anti‐tumour immunity is described above.

#### 2.3.3 Escape phase

In the escape phase (Dunn et al., 2002; Mittal et al., 2014), the tumour becomes more robust against immunological attack, becomes directly immunosuppressive, recruiting suppressor cells conferring further immunosuppression. Tumour resistance is increased through signal transducer and activator of transcription 3 (STAT3), apoptosis inhibiting proteins from the BCL2 family and by loss of expression of tumour antigen. Increased tumorigenesis may result from an increased inflammatory TME, epithelial mesothelial transition (EMT) and downregulation of Cadherin 1 (CDH1) (Mittal et al., 2014). There is downregulation of immunological attack, with suppression of NK cells (Pietra et al., 2012), Th‐1 cells and CTLs. The recruited immunosuppressive immunocytes from the bone marrow or periphery include tolerogenic DCs, regulatory T cells (Tregs), M2 macrophages and myeloid‐derived suppressor cells (MDSC). Effectors, many secreted/expressed by the tumour and also the above immunocytes, include immunosuppressive molecules, for example, indoleamine‐2,3‐dioxygenase (IDO), tryptophan‐2,3‐dioxygenase (TDO), arginase, the programmed death receptor ligand 1 (PDL1), cytotoxic T‐lymphocyte‐associated protein 4 (CTLA4), galectin‐1/3/9 and adenosine; immunosuppressive cytokines, for example, IL10, IL23; growth factors and colony stimulating agents (e.g. TGFB,VEGF, CSF1 and CSF2); and chemokines (e.g. CCL2, CXCL1 and CXCL5 (Michielsen et al., 2011).

#### 2.3.4 Immunosuppressive cells

Tolerogenic DCs have impaired antigen presentation capacity including to CTLs, with suppression of T‐cell proliferation and adaptive immune responses, (Tran Janco et al., 2015) and induce Tregs (Chen et al., 2008). As mentioned above. 1,25(OH)_2_D_3_ impairs DC maturation and survival, producing tolerogenic DC, an important facet of vitamin D_3_ immunoregulation (Adorini et al., 2004).

CD4+ Tregs are a highly immuno‐suppressive subset of CD4+ T cells, characterized by the expression of the master regulatory transcription factor FoxP3, (Fontenot et al., 2003) and promote tumour progression by suppressing effective antitumor immunity (Sakaguchi et al., 2010). Mechanisms include secretion of CTLA4, IL10, TGFB and granzyme/perforin, consumption of IL2 and adenosine production reviewed in (Sakaguchi et al., 2010). High infiltration of Tregs in tumours is associated with a poor prognosis in various types of cancers including MM (Fridman et al., 2012; Nishikawa & Sakaguchi, 2014). 1,25(OH)_2_D_3_ promotes the development of Tregs expressing CTLA4 and FOXP3 (Jeffery et al., 2009), the FOXP3 promoter containing a VDRE response element (Kang et al., 2012). In addition, vitamin D_3_ may indirectly promote preferential expansion of Tregs via IL2 and activation‐induced lymphocyte death (Hayes et al., 2015) and diverts Th‐17 differentiation towards Treg (Aranow, 2011), reviewed by Park & Pan (2015).

Suppressor γδT cells, reviewed by Zhao et al. (2018) comprise suppressive Vδ1 γδT cells and Vγ9Vδ2 T cells, polarized by immunosuppressive cytokines, including IL23, IL1B, IL15, IL17, IL4, IL10, IL36G and TGFB, in the TME, to FOXP3+ γδ Treg cells and γδ T17 cells. γδT regs have similar function to αβTreg cells, inducing DC and T‐cell senescence and suppressing naïve and effector T cells. γδT17 cells are a major source of IL17 in the TME resulting in increased angiogenesis with MDSC and neutrophil polymorph (PM) recruitment. Vδ1 γδT cells are particularly potent suppressors, promoting EMT via TGFB, impairing DC maturation and function, and are more powerful inhibitors of T4 cells than αβ Treg cells (Kuhl et al., 2009). Thus, γδT cells may have an anti‐cancer effect as described above or a pro‐cancer. A greater Vδ1:Vδ2‐ratio has a pro‐cancer effect and is increased by IL4 (Zhao et al., 2018). Evidence of a direct effect of vitamin D_3_ on suppressive γδT cells is lacking but vitamin D_3_ is known to upregulate FOXP3 as described above and a suppressive effect might be inferred from known effects on the immunosuppressive cytokines regulating Vγ9Vδ2 polarization and the Vδ1:Vδ2‐ratio. 1,25(OH)_2_D_3_ is known to upregulate the major suppressor cytokines IL4 (Boonstra et al., 2001), IL10 (Boonstra et al., 2001; Ragab et al., 2016) and TGFB (Cantorna et al., 1998), but also downregulate IL17 (Joshi et al., 2011) and the IL23 pathway (Faraji et al., 2016; Konya et al., 2018).

Type II NKT cells are typically associated with immunosuppression in animal cancer models (Nair & Dhodapkar, 2017). The mechanisms are downregulation of immunosurveillance and upregulation of immunosuppressive elements. Type II NKT cells suppress type I cells, CTLs, through IL13 production via IL4R and STAT6 axis, and conventional T cells inhibiting pro‐inflammatory function (Nair & Dhodapkar, 2017). The type II cell suppression predominates over type I cells when both are stimulated (Ambrosino et al., 2007). Type II cells tolerize myeloid DCs and induce‐MDSCs producing TGFB (mouse model fibrosarcoma). There are no reports of an effect of vitamin D_3_ on NKT type II cells in cancer, but it may induce immunosuppressive activity on Type 1 cells as described above.

M1 macrophage activity inhibits cell proliferation and causes tissue damage, whereas M2 macrophages promote cell proliferation and tissue repair (Bain & Mowat, 2014) and are more frequent in tumours (Mantovani et al., 2008). M2 macrophages promote angiogenesis, cell migration and intravasation (Lin & Pollard, 2007) and suppress adaptive immunity by PDL1 expression (Gibbons Johnson & Dong, 2017). M2 polarizing factors are hypoxia and acidity of the tumour microenvironment (Colegio et al., 2014), IL4, TGFB and IL10 and CSF2 (Su et al., 2014). Tumour‐associated macrophages (TAM) mainly have M2 polarisation and produce immunosuppressive cytokines such as IL10, TGFB and PGE2 and low levels of inflammatory cytokines (IL12, IL1B, TNF and IL6). Ability of TAMs to present tumour‐associated antigens is decreased as well as stimulation of the anti‐tumour functions of CTLs and NK cells. Vitamin D_3_ is reported to downregulate M1 and upregulate M2 macrophages in diabetic renal disease (Sloka et al., 2011; Zhang, Guo, Song, & Zhou, 2014), and a similar effect might be anticipated in cancer through its known upregulation of immunosuppressive cytokines.

MDSCs, recruited by tumour secreted CSF1 and CSF2, suppress T cells including CD8+, NK cells, DCs and macrophages. However, vitamin D_3_ opposes these effects by promoting differentiation of immature MDSCs into macrophages and DCs, reported in head and neck squamous cell carcinoma (Walsh et al., 2010). In this respect, a direct effect of vitamin D_3_ opposes suppression of anti‐cancer immunity. However, in an animal model with probable defective VDR signalling described below, MDSCs were increased (Cao et al., 2018).

#### 2.3.5 Effector mechanisms of the escape phase

IDO and TDO cause accumulation of immunosuppressive tryptophan catabolites, particularly kynurenine, resulting in suppression of NK cells (downregulation of activating receptors and granzyme content; Pietra et al., 2012), and antigen‐specific T‐cell responses, T‐cell apoptosis and increased proliferation of Tregs (Uyttenhove et al., 2003). 1,25(OH)_2_D_3_ has been shown to upregulate IDO resulting in increase of CD4 + CD25+ Tregs in multiple sclerosis (Correale et al., 2009) and 1,25(OH)_2_D_3_ induced IDO is a suggested mechanism for downregulation of Th‐1 priming and tolerogenic DC upregulation of Tregs (Gorman et al., 2010). Consequently IDO has been suggested as a general target of 1,25(OH)_2_D_3_ in the immune system (Dankers et al., 2016)

The programmed death receptor ligand 1 (PDL1), activates its receptor PD1 (member of CD28 family) on CD8 + T cells and represses TCR‐mediated activation and inhibits cell survival, proliferation and cytokine production (Parry et al., 2005). CTLA4, secreted by Tregs, blocks the co‐stimulatory signal from B7 on the APC and CD28 on the T4 lymphocyte, CTLA having a greater affinity for B7 molecules than CD28, thus inhibiting T4 effector function (Ribas & Wolchok, 2018). 1,25(OH)_2_D_3_ upregulates PDL1 and PDL2 and CTLA4 by direct transcriptional induction through the VDR and VDRE (Dimitrov et al., 2017) It has been suggested that elevated vitamin D_3_ signalling in humans could suppress anti‐tumour immunity via increased PDL1 expression. (Dimitrov et al., 2017) Extracellular adenosine is a physiological negative regulator of inflammation and immunity (Sitkovsky et al., 2004) and is largely produced from adenine nucleotides for example, ATP, by ecto‐5′‐nucleotidases, CD39 and CD73 (Eckle et al., 2007) Adenosine receptors, A2AR and A2BR are expressed in a wide variety of immune cells (Ohta & Sitkovsky, 2014). Effects include downregulation of T cells (including CD8+) (Linnemann et al., 2009); inhibition of T‐cell activation (Linnemann et al., 2009) proliferation and effector functions (Ohta et al., 2009), such as cytotoxicity and cytokine production (Raskovalova et al., 2007); inhibition of classical proinflammatory activation of APCs and induction of alternative activation (A2BR) (Ohta & Sitkovsky, 2014), resulting in APCs producing immunosuppressive molecules such as TGFB, IL10, arginase, IDO and COX2 (Novitskiy et al., 2008). Moreover, adenosine upregulates the number and activity of Tregs (Ohta et al., 2012; Ohta & Sitkovsky, 2014), and induces MDSCs (Ryzhov et al., 2011). 1,25(OH)_2_D_3_ upregulates adenosine production, via increased expression of CD39 and CD73 on CD4+ cells (Mann et al., 2015).

IL10 is a powerful tolerogenic agent, downregulating Th‐1 and Th‐2 responses, which may be secondary to a direct effect on monocyte–macrophages (Couper et al., 2008). IL10 downregulates MHC class II antigens, and co‐stimulatory molecules B71/B72 expression on macrophages. It activates STAT3 and induces enhanced expression of PD1 and PDL1 on DCs rendering them ineffective (Tran Janco et al., 2015), and is involved in polarizing γδT cells to tolerogenic cells (Zhao et al., 2018). Vitamin D_3_ is known to induce tolerogenic DCs and Tregs (Novitskiy et al., 2008; Sakaguchi et al., 2010) and to upregulate the transcription factor GATA3 and TH2 cells. (Boonstra et al., 2001), which are the sources of IL‐10. TGFB induces DC to stimulate Treg formation (Maldonado & von Andrian, 2010), polarizes FOXP3+ γδTreg cells from Vγ9/Vδ2 T cells (Casetti et al., 2009) and recruits TAM M2 macrophages (Byrne et al., 2008). There are reports of an inverse relationship between vitamin D_3_ and TGFB (Aschenbrenner et al., 2001; Isik et al., 2012). However, 1,25(OH)_2_D_3_ may co‐operate with TGFB, in the upregulation of immunosuppressive CD73 and FOXP3 expression and is reported to augment CD4+ expression of various TGFB associated molecules, and to increase bioactive TGFB (Mann et al., 2015).

**FIGURE 3:**
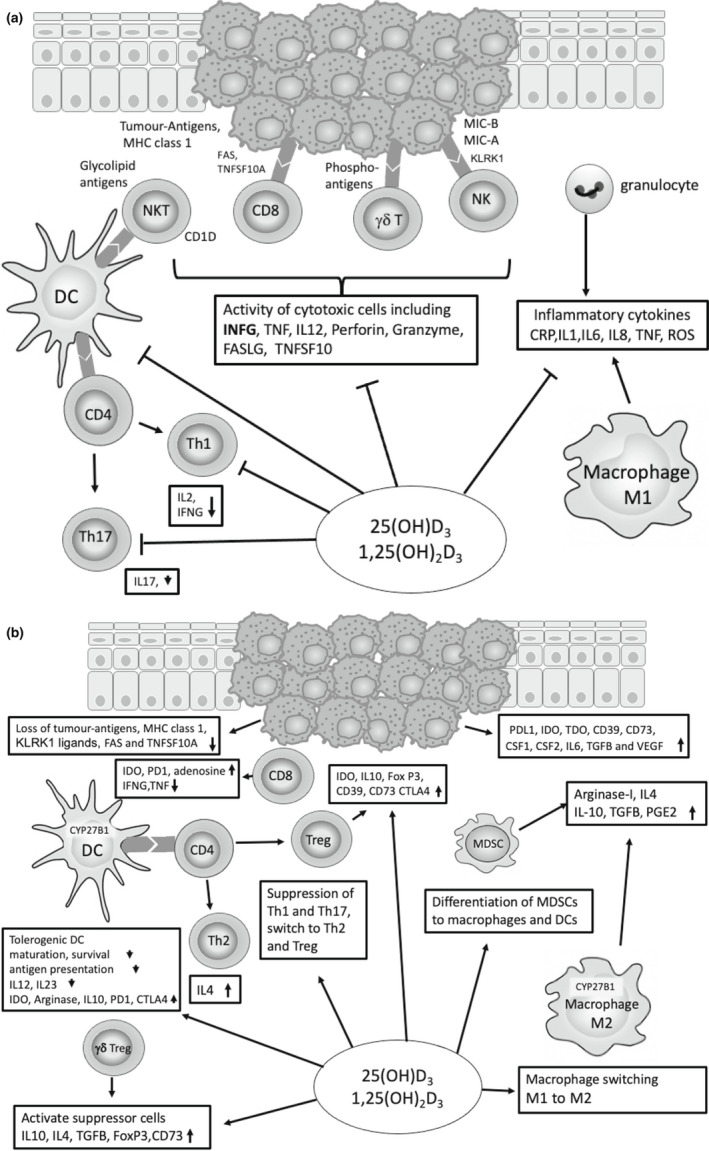
Vitamin D hydroxy derivatives have a direct effect on the immune response to melanoma. (a) Innate and acquired immunity in the elimination phase. The elimination phase involves both innate and acquired immunity. The tumour cells express the immune cell activating factors; KLRK1 ligands, phosphoantigens and MICA, MICB which activate γδT and NK cells, respectively; tumour glycolipids presented by CD1D activate NKT cells and tumour antigens in relation to MHC class 1 are recognized by CD8+ effector cells (CTLs). DCs increase the response by presenting tumour antigen to Th‐1 cells, NKT cells and CTLs. The activated immune cells secrete INFG, increasing tumour immunogenicity and upregulating DCs, Th‐1 cells, CTLs and macrophages, The activated immune cells kill tumour cells via apoptosis by inducing death signalling pathways of FAS and TNFSF10 and secretion of perforin and granzyme. IFNG can also mediate anti‐tumour effects by inhibiting tumour cell proliferation and angiogenesis. The activated immune cells and tumour cells can also recruit granulocytes and other immune cells by proinflammatory cytokines. The M1 macrophages and granulocytes secrete inflammatory cytokines, CRP, TNF, IL‐1, IL‐6, IL‐8 and ROS. The described effect of vitamin D_3_ in the elimination phase is to oppose the anti‐tumour immune response by downregulation of IFNG production and downregulated activity of DCs, NK cells, γδT cells, Th‐1 cells and CTLs. Vitamin D_3_ also downregulates M1 macrophages, decreasing Th‐17 cells inflammatory cytokine secretion. (b) Innate and acquired immunity in the escape phase. In the escape phase, the tumour evolves to be more resistant to immunological response, by losing immune cell activating factors and by recruiting suppressor cells conferring further immunosuppression. Tumour resistance is increased through STAT3, apoptosis inhibiting proteins from the BCL2 family, loss of death receptors FAS and TNFSF10A and by loss of surface antigens, MICA and MICB, KLRK1 ligands, tumour antigens and MHC class 1. The tumour expresses immunosuppressive molecules, PD‐L1, IDO, TDO and adenosine producing enzymes (CD39 and CD73) and secretes growth factors for example, GCSF, GMCFS and VEGF. The recruited immunosuppressive immunocytes include, tolerogenic DCs, Tregs, MDSCs, suppressor γδTregs, Type II NKT cells and M2 macrophages. These may similarly express IDO (tolerogenic DCs, MDSCs, Tregs and M2 macrophages), CD39 and CD73 (Tregs, which also secrete CTLA4) and arginase (tolerogenic DCs, MDSCs and M2 macrophages) and secrete immunosuppressive cytokines, IL‐10, TBFB. The resulting effect on the anti‐tumour immunity is downregulation of NK cells (IDO), DC antigen presentation (CTLA4), switch Th1 to Th2 cells (IDO, adenosine, IL‐10) and CTLs (IDO. PD‐1, adenosine). 1,25(OH)_2_D_3_ can upregulate IDO, PDL‐1 expression, CTLA4, adenosine production, via increased expression of CD39 and CD73 on CD4+ cells, and secretion of immunosuppressive cytokines, IL‐10, TGFB, IL‐4. Mature macrophages and DCs can also express the enzyme 1α‐hydroxylase (CYP27B1) allowing intracrine and paracrine synthesis of 1,25(OH)_2_D_3_ suppressing maturation of DCs, switching M1 to M2 macrophages and enhancing a tolerogenic immune response. Therefore, the effect of 1,25(OH)_2_D_3_ on suppressive immunocytes is to generate tolerogenic DCs (via impaired DC maturation), CD4+ Tregs (CTLA4, IL10, TGFB, adenosine and FOXP3), and suppressor γδT cells (suppressor cytokines). 1,25(OH)_2_D_3_ also differentiates MDSCs to DCs and macrophages. The anticipated effect on anti‐tumour immunity is accentuation of the tumour induced suppression of DCs, NK cells, Th‐1 and CTL responses

Thus, in the absence of tumour VDR signalling, many of the reported immunosuppressive effects of vitamin D_3_, reported in a non‐tumour context, may be relevant to tumour immunity as they would apparently oppose immune suppressive effects on the tumour in the elimination phase, tip the balance in the equilibrium phase towards tumour expansion by downregulating anti‐tumour immunity and potentially amplify immunosuppression in the escape phase, having overlapping immunosuppressive activities with some of those of the escape phase. These include the development of immunosuppressive immunocytes, tolerogenic DCs, Tregs and M2 macrophages but possibly not MDSCs and mechanistic similarities, involving IDO, PDL1, CTLA, adenosine, IL10 and TGFB. Figure 3. shows a summary of the direct influence of vitamin D influence on innate and adaptive immunity which may affect the immune response to cancer in the elimination (Figure 3a) and escape phases (Figure 3b) of immunoediting in cancer.

### 2.4 Angiogenesis

Angiogenesis is necessary for local tumour invasion and metastasis. The VDR is expressed in endothelial cells and vascular smooth muscle cells and vitamin D_3_ promotes angiogenesis and VEGF secretion (Cardus et al., 2009; Grundmann et al., 2012). However, in the context of tumours, there is evidence of an anti‐angiogenic effect of vitamin D_3_ (Ma et al., 2011). In vivo tumour‐cell induced angiogenesis is reportedly inhibited by 1,25(OH)_2_D_3_ and retinoids synergistically (Majewski et al., 1993). Furthermore, in a colon cancer model, 1,25(OH)_2_D_3_ inhibited angiogenesis, which was associated with reduced VEGF expression in tumours (Iseki et al., 1999).

These opposing effects of vitamin D_3_ might be reconciled by the postulate of tumour VDR inhibiting a pro‐angiogenic factor secreted by the tumour. Loss of tumour VDR would leave a direct vascular effect of vitamin D_3_ unopposed. This would be analogous to the effects of vitamin D_3_ on immunity as described above. Furthermore, Wnt beta‐catenin signalling is known to promote angiogenesis (Chen et al., 2009).

## 3 THE REPORTED EFFECT OF VITAMIN D_3_ IN CANCER

### 3.1 Animal studies—the effect of vitamin D3/1,25(OH)2D3 or vitamin D3 analogues on cancer xenographs

Several experimental studies with explanted human or mouse cancer tissue have shown that Vitamin D_3_ is associated with inhibition of tumour growth (Krishnan et al., 2013; Milczarek et al., 2013; Ooi et al., 2010; Swami et al., 2012; Williams et al., 2016) and metastasis. However, there is also experimental evidence of vitamin D_3_ promoting tumour progression with metastasis and decreased survival (Anisiewicz et al., 2018; Cao et al., 2018). It is notable that in the studies showing a beneficial effect, the malignant cells were ‘sensitive’ (in terms of inhibition of proliferation) to the direct action of vitamin D_3_ and/or immune deficient models were used (Pawlik et al., 2018; Zhang, Guo, Zhang, et al., 2014). In animals showing a deleterious effect, the tumour was not sensitive in vivo nor in vitro (Pawlik et al., 2018). In these animals, transcription was most prominently upregulated in genes of Tregs and Th‐2 cells. In a further study, vitamin D administration was associated with a decrease in Th‐1 cells, an increase in MDSCs and decreased transcription of INFG with increased transcription of TGFB (Cao et al., 2018). Thus, sensitivity to growth inhibitory effects of vitamin D_3_, which would imply effective tumour VDR signalling, was associated with a beneficial effect but a deleterious effect, with immunosuppression, if not.

### 3.2 Observational studies

#### 3.2.1 Cancer development

Prediagnostic vitamin D_3_ status has an undeniably important protective effect on the development and subsequent progression of a variety of cancers, comprehensively reviewed by Grant (2018). The evidence is largely epidemiological based upon an inverse relation of incidence and/or outcome of a variety of carcinomas with indices of solar UVB exposure (Fleischer & Fleischer, 2016; Garland & Garland, 1980; Garland, Garland, et al., 1990; Garland, White, et al., 1990; Grant, 2002; Zamoiski et al., 2016) including latitude (Grant, 2007) and also modifying issues of dark skin (Grant & Peiris, 2012) and outdoor occupation (Grant, 2012; Pukkala et al., 2009).

#### 3.2.2 Vitamin D levels and established cancer

A majority of observational studies of post‐diagnosis 25(OH)D_3_ serum levels have shown an inverse relation with progression in a variety of cancers (Vaughan‐Shaw et al., 2017) including MM (Newton‐Bishop et al., 2009; Nurnberg et al., 2009). This might be expected early post diagnosis, these levels being a reflection of prediagnosis levels which would have a formative effect on cancer development, and hence, an effect on cancer progression as found in the prospective studies cited above. Supportive of this, a study which measured serum 25(OH)D_3_ soon after diagnosis and also assessed previous sun exposure, through patient diaries, concluded that the ‘measured serum 25(OH)D_3_ levels not only reflected the recent sun exposure, but could also be considered to be representative for a period of at least several years’ (Nurnberg et al., 2009). The post‐diagnosis findings have been interpreted (Newton‐Bishop et al., 2009; Nurnberg et al., 2009) as vitamin D_3_ administration having a beneficial effect on established cancer. This is likely to be valid for early developing cancers but, in more advanced cancer, we believe this concept should be tempered by VDR status as discussed above. There are few reports of 25(OH)D_3_ levels later during follow‐up. One study found that, compared with initial 25(OH)D_3_ levels, both decreased and increased later levels were associated with worsened prognosis, which prompted the authors to caution against widespread use of vitamin D_3_ supplementation in melanoma patients (Saiag et al., 2015). A further study found that blood levels taken after resection of regional nodes, sometimes years after initial diagnosis in stage III MM patients, had no relationship with prognostic indices or survival (Lipplaa et al., 2018).

### 3.3 Interventional studies

#### 3.3.1 Vitamin D supplements and development and subsequent progression of cancer

Randomized controlled trials on vitamin D supplementation, reviewed by Keum et al. (2019), have shown a variable effect on cancer incidence but a protective effect with larger dose and a more consistent protective effect on subsequent mortality.

#### 3.3.2 1,25(OH)2D3 or vitamin D3 analogue supplements in established cancer

A trial of large dose vitamin D_3_ in advanced MM was documented in 2014 (Saw et al., 2014) but results are still awaited. A placebo‐controlled trial on vitamin D_3_ supplementation (100,000 IU every 50 days for 3 years) for resected Stage II MM patients (MelaViD trial) was posted in 2010 but was terminated in 2017 because of inadequate recruitment (150 patients) and no results were reported (De Smedt et al., 2017). A phase 2 study high‐ vs low‐dose vitamin D_3_ plus standard chemotherapy in 139 metastatic colon cancer (CRC) patients showed a significant (*p* = .04) advantage in progression free survival (PFS) of high‐dose vitamin D_3_ (Ng et al., 2019); result of a confirmatory phase 3 trial is awaited. However, a study of 2000 IU/d cholecalciferol vs placebo in patients with alimentary cancer, including CRC, showed no significant effect on 5‐year relapse‐free survival, (Urashima et al., 2019) and a similar study lasting two years following diagnosis, in metastatic CRC, showed no benefit to overall survival (Antunac Golubic et al., 2018). A retrospective, single institution, study of vitamin D_3_ supplementation (‘low dose’) in non‐metastatic HER2+ breast cancer reported a prolongation of disease‐free survival (Zeichner et al., 2015). However, the same study showed a deleterious effect in larger tumours. Larger or deeper tumours are likely to be more advanced and thus, VDR signalling less likely to be intact (Hutchinson et al., 2018). A pilot study of 16 patients with head and neck SCC being treated with 1,25(OH)_2_D_3_ during the 3‐week interval between cancer diagnosis and surgical treatment (3 cycles of 4 μg of 1,25(OH)_2_D_3_ for each of 3 sequential days, followed by 4 days) showed a prolongation of time to recurrence in the treated group (*p* = .04) (Walsh et al., 2010). No further results appear to have been published. A study in low‐grade prostate cancer given high dose vitamin D_3_ for a year showed improvement compared with historical controls (Marshall et al., 2012). In advanced malignancy, a number of uncontrolled studies have shown modest or no measurable improvement in advanced prostate, pancreatic and hepatic cancer (Beer, Lemmon, et al., 2003; Dalhoff et al., 2003; Evans et al., 2002; Liu et al., 2002; Schwartz et al., 2005) and similarly 1,25(OH)_2_D_3_ combined with carboplatin in prostate cancer (Beer et al., 2004; Flaig et al., 2006). High‐dose 1,25(OH)_2_D_3_ plus docetaxel showed promising results in prostate cancer (Beer, Eilers, et al., 2003) and was followed by a controlled trial of docetaxel with or without high dose 1,25(OH)_2_D_3_, which just failed to show a significant effect of the 1,25(OH)_2_D_3_ arm (Beer et al., 2007). This was followed by a large phase 3 (ASCENT) study which included dexamethasone in both arms and prednisolone in the placebo arm. This trial was halted because of excess deaths in the 1,25(OH)_2_D_3_ arm (Scher et al., 2011). Thus, there is evidence of some beneficial effect of vitamin D_3_. particularly in early disease but also of a deleterious effect, particularly in advanced disease.

## 4 COMMENT

There is evidence for a beneficial effect of vitamin D_3_ in the processes involved in cancer, with the suppression of growth and inflammation, enhancement of anti‐tumour immunity and suppression of angiogenesis. However, there are differences between the reported effects of vitamin D_3_ in cancerous and non‐cancerous contexts on immunity and angiogenesis. VDR signalling is of obvious importance in tumour cells but also in inflammatory cells, immunocytes and angiocytes. With loss of tumour cell VDR signalling, vitamin D_3_ signalling in other cells in the TME continues and may gain significance. The reported beneficial effect of vitamin D_3_ on tumour immunity (Muralidhar et al., 2019) would appear dependent on tumour cell VDR signalling. In the absence of tumour VDR signalling, some beneficial effects of vitamin D_3_ that is, the suppression of inflammation and possibly suppression of MDSCs, would be expected to continue but deleterious effects would seem likely to emerge, with loss of tumour growth suppression, suppression of anti‐tumour immunity and possibly upregulation of tumour angiogenesis. Anti‐tumour immunity may be particularly important. In cancers, such as MM, where tumour VDR enhances anti‐tumour immunity, loss of tumour VDR signalling might be expected to result in opposition of the elimination phase, tipping the equilibrium phase in favour of tumour progression and enhancement of the escape phase by the direct action of vitamin D_3_ on immunocytes.

Observational studies of early post diagnosis 25(OH)D_3_ levels have shown a protective effect on progression in a number of cancers. (Newton‐Bishop et al., 2009; Nurnberg et al., 2009; Vaughan‐Shaw et al., 2017) However, these levels are a likely reflection of prediagnosis levels which are known to have a formative effect on cancer development and progression. Levels taken later in established cancer are infrequently reported and have shown varying associations including a deleterious effect. In animal models, where tumour VDR signalling was apparently defective, vitamin D_3_ administration decreased survival and increased metastases, associated with downregulation of Th‐1 cells and INFG gamma and upregulation of MDSCs and TGFB (Anisiewicz et al., 2018; Cao et al., 2018) and upregulation of transcription of Tregs and Th‐2 cells (Pawlik et al., 2018). In advanced human disease (a likely marker of impaired cancer cell VDR signalling, nuclear VDR levels being inversely related to tumour progression (Brozyna et al., 2011; Hutchinson et al., 2018; Kivineva et al., 1998; Kure et al., 2009; Matusiak et al., 2005; Menezes et al., 2008; Salehin et al., 2012), a number of uncontrolled studies of high‐dose vitamin D_3_ have shown modest or no measurable improvement in advanced prostate, pancreatic and hepatic cancer (Beer, Lemmon, et al., 2003; Dalhoff et al., 2003; Evans et al., 2002; Liu et al., 2002; Schwartz et al., 2005). There is therefore no obvious evidence that vitamin D_3_ is beneficial in these cancers. Moreover, a deleterious effect could be masked if in some of the tumours, VDR signalling remained intact producing a marked beneficial effect. In addition, in a large‐controlled study of docetaxel and dexamethasone with or without high dose 1,25(OH)_2_D_3_, there were excessive deaths in the treated arm (Scher et al., 2011). Unfortunately, the results of some studies started several years ago have not been reported.

Thus, 25(OH)D_3_ levels taken at diagnosis appear a questionable method of assessing likely vitamin D_3_ response in later disease, and there are theoretical and demonstrated risks, from animal and clinical studies, of vitamin D_3_ administration in advanced cancer. Critical factors are the integrity of tumour cell VDR signalling and perhaps dosage. The NICE recommendation (NICE, T.N.I.f.H.a.C.E, 2015) is vitamin D_3_ administration to MM patients with deficient serum levels. This is given without the reference to tumour VDR signalling status, and there is no warning about using high dose vitamin D_3_. Unfortunately, there is no accepted routine method of assessing VDR signalling. Indicators of effective VDR signalling are higher levels of VDR mRNA (Muralidhar et al., 2019), predominantly nuclear VDR (Kivineva et al., 1998; Kure et al., 2009; Matusiak et al., 2005; Menezes et al., 2008; Salehin et al., 2012) and at a clinical level early as opposed to advanced disease.

More work is needed on assessing the integrity of tumour VDR signalling in cancer and trials are necessary to assess the safety of vitamin D_3_ supplementation, including small dose, in tumours with defective VDR signalling. A further treatment possibility is to rectify defective VDR signalling as recently suggested (Muralidhar et al., 2019), and one possibility is through MAPK inhibition (Hutchinson et al., 2018).

## CONFLICT OF INTEREST

The authors declare no conflict of interest for preparing this manuscript.

## Data Availability

Data sharing is not applicable to this article as no new data were created or analyzed in this study.
